# Predicting Radiotherapy Outcomes with Deep Learning Models Through Baseline and Adaptive Simulation Computed Tomography in Patients with Pharyngeal Cancer

**DOI:** 10.3390/cancers17213492

**Published:** 2025-10-30

**Authors:** Kuo-Chen Wu, Shang-Wen Chen, Yuan-Yen Chang, Yao-Ching Wang, Ying-Chun Lin, Chao-Jen Chang, Zong-Kai Hsu, Ruey-Feng Chang, Chia-Hung Kao

**Affiliations:** 1 Graduate Institute of Biomedical Electronics and Bioinformatics, National Taiwan University, Taipei 10617, Taiwan; 2 Artificial Intelligence and Robotics Innovation Center, China Medical University Hospital, Taichung 404327, Taiwan; 034808@tool.caaumed.org.tw (C.-J.C.);; 3 School of Medicine, China Medical University, Taichung 40402, Taiwan; 4 School of Medicine, Taipei Medical University, Taipei 11031, Taiwan; 5 Department of Radiation Oncology, China Medical University Hospital, Taichung 404327, Taiwan; 6 Department of Computer Science and Information Engineering, National Taichung University of Science and Technology, Taichung 404336, Taiwan; 7 Department of Biomedical Imaging and Radiological Science, China Medical University, Taichung 40402, Taiwan; 8 Department of Computer Science and Information Engineering, National Taiwan University, Taipei 10617, Taiwan; 9 Graduate Institute of Biomedical Sciences, School of Medicine, College of Medicine, China Medical University, Taichung 40402, Taiwan; 10 Department of Bioinformatics and Medical Engineering, Asia University, Taichung 413305, Taiwan; 11 Department of Nuclear Medicine and PET Center, China Medical University Hospital, Taichung 404327, Taiwan

**Keywords:** computed tomography, deep contrastive learning, pharyngeal cancer, adaptive radiotherapy, treatment outcome

## Abstract

**Simple Summary:**

This study developed a deep learning model to predict treatment outcomes in pharyngeal cancer patients undergoing radiotherapy. We analyzed 162 patients with oropharyngeal or hypopharyngeal cancer who received definitive radiotherapy between 2008 and 2020. The model utilizes both baseline and adaptive radiation therapy (ART) simulation CT images to forecast the risk of local recurrence, nodal relapse, and distant metastasis. Using a deep contrastive learning framework with a merged ensemble approach, the model achieved area under the curve values of 0.773, 0.747, and 0.793 for predicting these three endpoints, respectively, with corresponding accuracies of 72.4%, 74.7%, and 75.7%. While these results demonstrate promising predictive capability, external validation with multi-center datasets is essential to confirm the model’s generalizability and robustness across different patient populations and imaging protocols.

**Abstract:**

**Background/Objectives**: The implementation of adaptive radiation therapy (ART) is increasingly becoming widely available in the clinical practice of radiotherapy (RT). For patients with pharyngeal cancer receiving RT, this study aimed to develop a deep learning (DL) model by merging baseline and ART simulation computed tomography (CT) images to predict treatment outcomes. **Methods**: Clinical and imaging data from 162 patients of newly diagnosed oropharyngeal or hypopharyngeal cancer were analyzed. All completed definitive treatment and their baseline and ART non-contrast simulation CTs were utilized for training. After augmentation of the CT images, a deep contrastive learning model was employed to predict the occurrence of local recurrence (LR), neck lymph node relapse (NR), and distant metastases (DM). Receiver operating characteristic curve analysis was conducted to evaluate the model’s performance. **Results**: Over a median follow-up period of 34 months, 53 (32.7%), 36 (22.2%), and 23 (14.0%) patients developed LR, NR, and DM, respectively. Following the integration of prediction results from baseline and ART simulation CTs, the area under the curve for predicting the occurrence of LR, NR, and DM reached 0.773, 0.747, and 0.793. At the same time, the accuracy for the three endpoints was 72.4%, 74.7%, and 75.7%, respectively. **Conclusions**: For patients with pharyngeal cancer ready to receive RT-based treatment, our proposed models can predict the development of LR, NR, or DM through baseline and ART simulation CTs. External validation needs to be conducted to confirm the model’s performance.

## 1. Introduction

Adaptive radiation therapy (ART) has become increasingly accessible in clinical settings throughout the radiotherapy (RT) process [1]. By utilizing timely simulation computed tomography (CT), ART can potentially minimize treatment-related toxicities or escalate target doses [1,2]. Head and neck squamous cell carcinomas (HNSCC), prevalent malignancies globally [3], are characterized by heterogeneity due to their origins in different anatomical regions such as the oral cavity, pharynx, or larynx. RT, whether as an adjuvant or a primary treatment, is frequently used to manage these cancers [4]. As a result, ART is commonly applied to HNSCC patients during their treatment [2,5]. Recent studies have investigated optimal timing for ART implementation. found that the 3rd week was optimal for re-planning in 10 OARs, with 2 re-plannings sufficient to maintain dose deviation below 3 Gy [6].

Except for some biomarkers [7], most treatment protocols for HNSCCs are grounded in the anatomical scope of the disease or its clinical stage. Although tumors exhibit biological diversity [3], often influencing how they respond to particular treatments [4], these molecular variations are seldom factored into managing this cancer. Radiomics, which quantifies tumor phenotypes, has recently gained attention for predicting treatment outcomes. However, the radiomics workflow can be influenced by several elements that may compromise the robustness and transferability of models across diverse patient populations and institutions [8].

Recent research has demonstrated the efficacy of image-based deep learning (DL) frameworks in identifying distinct patient subpopulations and personalizing RT dosages [9]. For patients with locally advanced HNSCC originating in the oropharynx or hypopharynx, chemoradiotherapy (CRT) for organ-preserving RT is typically recommended. However, there is a notable scarcity of DL-based research investigating the predictive performance for clinical outcomes in this context [10,11,12]. Given that a patient’s susceptibility to tumor recurrence is crucial in determining an appropriate treatment plan, it is essential to develop an effective prognostic model when discussing treatment options. We postulated that combining baseline and ART simulation CT data could enhance predictive accuracy and robustness by addressing potential overfitting issues associated with single-model architectures. Integrating imaging biomarkers may improve RT-based treatment outcomes for these patients. Our research may provide oncologists with earlier opportunities to evaluate alternative treatment modalities, implement radiation dose escalation protocols, or initiate innovative combination therapies for high-risk patients.

## 2. Materials and Methods

### 2.1. Study Population

This retrospective study analyzed data from patients freshly diagnosed oropharyngeal or hypopharyngeal squamous cell carcinoma treated with definitive CRT or RT for organ preservation between January 2008 and December 2020. The presence of neck lymph node metastases was determined using either [18F] Fluorodeoxyglucose Positron Emission Tomography/Computed Tomography (18F-FDG PET/CT) or CT scan. On CT imaging, lymph nodes were considered metastatic lesions if their shortest axis diameter was more significant than 1 cm.

Patients were included if they met the following criteria:

1. Completed the prescribed RT or CRT and were followed for at least 6 months or until death.
2. Underwent a comprehensive staging process including physical examination, laryngoscopy, tumor biopsy, chest radiography, and either a CT scan of the neck or 18F-FDG PET/CT.
3. They were classified as having American Joint Committee on Cancer (AJCC) stage III to IVB disease, with a clear distinction between the primary tumor and nodal involvement.
4. Had a planned adaptive radiotherapy (ART) simulation CT scan performed approximately 4 to 5 weeks after the start of radiation therapy.

The local institutional review board approved this study [certificate numbers: CMUH106-REC3-119(CR-6)].

### 2.2. Study Endpoints and Design

**Figure 1:**
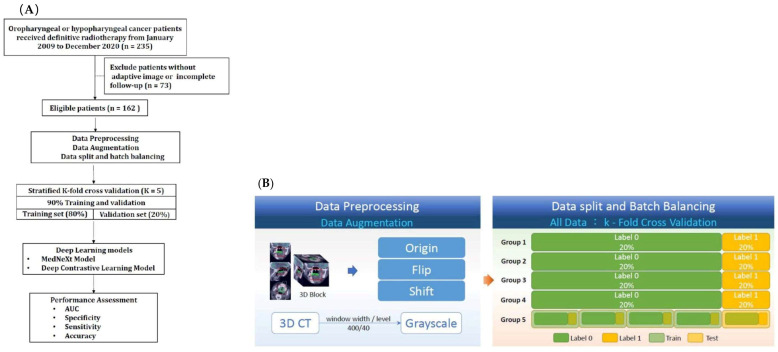
To minimize the probability of a combination of all zeros or all ones in a batch size, a Batch Balance Wrapper framework was applied in this study. All data were randomly displaced to the index according to label 0 or label 1. As a result, the ratio of all labels could be balanced. (**A**) The workflow of this study. (**B**) Data preprocessing, augmentation, split and batch balancing.

This research centered on three pivotal clinical events with significantly impacting survival: local recurrence (LR), neck lymph node relapse (NR), and distant metastasis (DM). LR and NR were characterized by either persistent or newly developing disease, verified via laryngoscopy, biopsy, or serial imaging assessments. The determination of DM hinged upon the detection of recurrent tumors located beyond the initial radiation treatment fields. The study’s overall process is visually outlined in Figure 1A.

The rationale for utilizing both baseline and ART simulation CT images was to capture the dynamic changes occurring during the course of RT. Baseline CT provided information on the initial tumor characteristics, such as volume and heterogeneity. The ART simulation CTs reflected the early response of tumor to therapy and any accompanying anatomical modifications. By integrating features from both time points, we aimed to achieve more accurate prediction of treatment outcomes compared to that of using either time point alone.

### 2.3. Simulation CT Image Acquisition

To ensure positioning consistency, each patient was immobilized supine using a thermoplastic mask during CT simulation. Images were acquired using a HiSpeed NX/I CT scanner (GE Healthcare, Orlando, FL, USA). The scan range extended from the skull base to 2 cm inferior to the sternum, with a slice thickness of 3 mm. Contrast agents were not used during image acquisition.

### 2.4. Tumor Volume Delineation

As outlined previously [13], the CT images from the picture archiving and communication system were transferred to a commercial planning system (Eclipse Version 8.1, Varian Medical System Inc., San Jose, CA, USA). Radiation oncologists subsequently delineated the pretreatment gross tumor volume of the primary tumor (GTVp) and the metastatic lymph node volume (GTVn). The volumes of all tumors were determined by outlining the lesion on each image where it was visible. No effort was made to differentiate the tumors from any related edema. To ensure accuracy and minimize interobserver variability in tumor delineation, we implemented a multi-reviewer protocol. Each patient’s tumor volumes were independently contoured by two radiation oncologists using the Eclipse planning system. Measurements with less than 10% variance were averaged, while greater discrepancies required re-evaluation by a third radiation oncologist to resolve differences. This quality control process was applied to both baseline and ART simulation CT images. The similar contouring scheme was applied to ART CT simulation images.

### 2.5. Data Preprocessing

Researchers defined the anatomical coordinates for the region of interest (ROI). The initial CT images were transformed from a format of 512 × 512 depth matrices. The three-dimensional (3D) blocks, extracted from CT images as input data for training, were composed of a dimension of 128 × 128 × 48 pixels. The ROIs, which represented the anatomical sites of primary tumors or metastatic neck lymph nodes, were not always defined as continuous regions. This discontinuity might provide more biological information to reflect the tumor heterogeneity.

### 2.6. Data Augmentation

To mitigate the risk of overfitting, we implemented a random modification of the 3D ROIs as part of the augmentation process. Image augmentation was performed using random flipping or transformation in approximately 20% of the random area of the ROIs. Then, CT images were converted to a grayscale format by adjusting suitable window levels (width/level of 400/40). In addition, the contrast of CT images fluctuated up and down within a fixed scope. Random tuning of the contrast was carried out to enhance the robustness of the image’s qualities. By reducing the dependence on setting parameters during imaging processing, the input data can mimic the situation in the real world. Moreover, the anatomical and biological characteristics derived from 3D blocks of a tumor could be captured through a random change in the 3D ROIs and tunning of the contrast.

### 2.7. Data Split and Batch Balancing

The study cohort was divided into five subsets to evaluate the model’s robustness, each maintaining a similar distribution of patients with or without the defined clinical events (LR, NR, DM). A 5-fold cross-validation approach was utilized, where each subset served as the test set once, while the remaining four were combined to create the training dataset. This resulted in an 8:2 ratio of training to testing data across all imaging data. The predictive parameters reported represent the mean values obtained from this cross-validation process (Figure 1B).

To ensure a balanced representation of different outcomes within each training batch and expedite model convergence, a Batch Balance Wrapper framework was implemented. This framework minimized the likelihood of batches containing exclusively one outcome (positive or all negative for the event being predicted). Data points were randomly assigned to indices based on their outcome label (0 or 1), ensuring a balanced representation of each outcome within each batch during training (Figure 1B). Within this framework, for each specific prediction task, data points were assigned a binary outcome. The label of ‘0’ represented the absence of the event, and ‘1’ signified the presence of the event.

### 2.8. Model Training and Optimization

Deep contrastive learning (DCL) is a representation learning technique that aims to learn a feature space where similar samples are close to each other while dissimilar samples are far apart [14]. Traditional DCL is mainly applied in unsupervised learning, but we extended it to a supervised learning task for classification by introducing supervised contrastive loss, as detailed in Appendix A.1.

**Figure 2:**
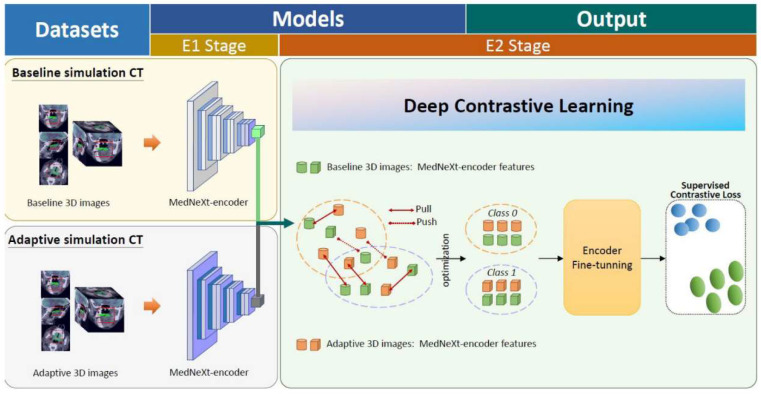
Merged ensemble model. The training process was structured into two distinct phases: E1 (individual model optimization) and E2 (merged ensemble model). The E1 phase employed a deep learning framework based on the MedNeXt architecture, in which the classification tasks were carried out. This implementation, utilizing PyTorch (2.5.0), focused exclusively on the encoder segment of the network. Key architectural modifications included the integration of a global average pooling layer preceding the softmax function and the implementation of a temperature scaling mechanism (T = 0.01) to enhance model generalization. The E2 phase involved the development of a merged ensemble model, aggregating prediction results from the E1 phase. The ensemble approach was designed to leverage the complementary information captured by the individual models, potentially enhancing the overall prediction accuracy and stability of our framework. This dual-model approach was conceptualized to capture temporal variations in prognostic indicators throughout the treatment course. Red lines indicates the cancer data extraction range; green lines indicates the tumor range.

The training regimen was divided into two sequential phases: E1, focused on optimizing individual models, and E2, dedicated to developing a merged ensemble model. During the E1 phase, each model underwent independent training and fine-tuning. This was performed to assess each model’s baseline and ART-simulated CT prediction performance across designated endpoints. This individual optimization step was critical, ensuring that each constituent model reached peak performance before integration into the ensemble. The subsequent E2 phase centered on the creation of the merged ensemble model. This ensemble model was designed to synthesize the predictions generated by the individual models from E1, ultimately determining the final prediction, as depicted in Figure 2. This ensemble approach was implemented to enhance the accuracy and robustness of the projections, mitigating the risk of overfitting that can occur with reliance on a single model.

### 2.9. Postprocessing

In this study, a threshold of 0.5 was adopted to apply the loss function. When we needed to establish a definitive cutoff for classifying results into positive or negative categories, we employed the optimal threshold values identified from the receiver operating characteristic (ROC) curves. This approach was taken to maximize the sensitivity and specificity of the predictive model’s loss function. The ultimate prediction scores were derived by aggregating the outcomes obtained from baseline and ART-simulated CT scans.

### 2.10. Treatment

Adhering to our previously established protocol [13], patients were treated with a sequential intensity-modulated RT (IMRT) approach. A daily dose of 1.8 Gray (Gy) was administered, leading to a cumulative dose ranging between 68.4 and 72 Gy (median of 70.2 Gy). Two distinct clinical target volumes (CTVs) were delineated according to risk stratification. CTV1 included the primary gross tumor volume (GTVp), any metastatic lymph nodes, and the adjacent areas deemed at immediate risk. CTV2 comprised ipsilateral or contralateral N0 regions where the potential for microscopic disease was considered significant. The initial treatment phase involved delivering 50.4–54.0 Gy to CTV1 and CTV2. Following this, a 16.2–21.6 Gy boost was directed to CTV1 in the second phase. Consequently, the median total doses received were 70.2 Gy for CTV1 and 50.4 Gy for CTV2. The median treatment duration was 55 days.

The majority of patients underwent concurrent chemotherapy using cisplatin, given at a dosage of 80–100 mg/m^2^ on days 1, 22, and 43 of the RT regimen. A subset of patients was treated with a regimen combining cetuximab and RT, starting with an initial dose of 400 mg/m^2^, followed by 250 mg/m^2^ for subsequent doses. A portion of the cohort received RT as a standalone therapy.

### 2.11. Follow-Up

Following the completion of therapy, patients underwent regular follow-up assessments. These were conducted bimonthly for the initial two-year period, then at 3–4 months intervals subsequently. Each follow-up visit included a comprehensive physical examination and laryngoscopic evaluation. Additionally, neck CT scans were performed every 4–6 months during the first two years post-treatment. Treatment failure was determined based on findings from laryngoscopy, neck CT, or 18F-FDG PET/CT examinations. In cases where persistent tumors or local recurrences were identified, salvage surgery was proposed as a treatment option, provided it was technically viable.

### 2.12. Statistical Analysis

We employed the area under the receiver operating characteristic (ROC) curve (AUC) as a primary metric to evaluate predictive performance. Furthermore, sensitivity (SE), specificity (SP), and accuracy were assessed to provide a more comprehensive performance profile. In addition, the prediction performance from clinical stage and the GTVs were analyzed for comparison. All statistical computations were performed using IBM SPSS Statistics (version 26, IBM Cooperation, Armonk, NY, USA).

## 3. Results

### 3.1. Patient Characteristics and Treatment Outcome

One hundred and sixty-two patients who met the selection criteria were included. As summarized in Table 1, 23, 125, and 14 patients were categorized as stage III, IVA, and IVB disease. The median age was 53 years (37–82 years).

ART simulation CTs were acquired during the fourth to fifth week of treatment (median: 29 days; range: 22–36 days post-RT initiation). This timing was selected to capture early tumor response while maintaining sufficient opportunity for treatment plan adaptation based on anatomical and volumetric changes.

**Figure 3:**
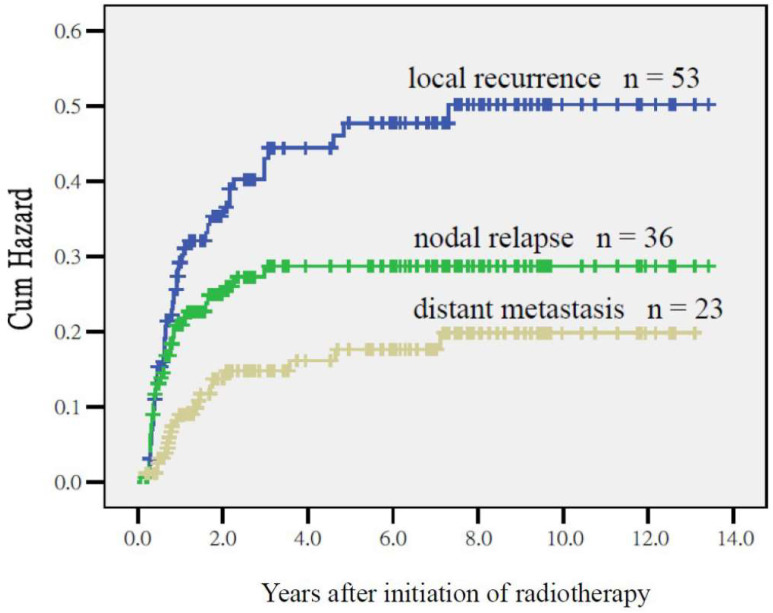
The hazard of local recurrence, nodal relapse, and distant metastasis.

After a median follow-up duration of 34 months (range, 3 to 158 months), 53 (32.7%) and 36 (22.2%) patients in this cohort developed LR and NR, whereas 23 patients (14.2%) were confirmed to experienced DM by their consecutive images. The 3-year overall survival rate was 49%. Figure 3 illustrates the temporal distribution of treatment failures. The majority of local recurrence, nodal relapse, and distant metastasis events were observed within the first three years following treatment completion, with peak incidence occurring during the second year.

### 3.2. Patient-Based Prediction

The predictive performance of our models for LR, NR, and DM, evaluated across five subsets of the training cohort, is summarized in Table 2, Table 3 and Table 4, respectively. This comprehensive evaluation ensures the robustness of our findings. Regarding the individual prediction performance, generally, the values from baseline CTs demonstrated a superior predictive ability across the three endpoints when compared to that of ART CT images.

**Figure 4:**
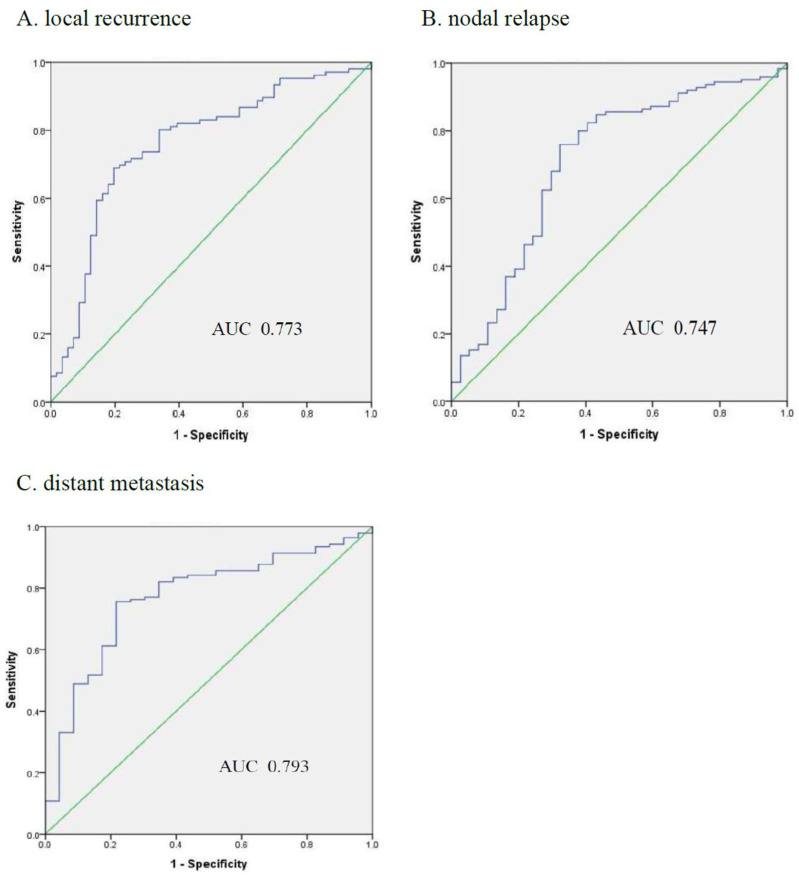
ROC curves of prediction performance by the merged ensemble model for three clinical endpoints ((**A**): local recurrence; (**B**): nodal relapse; (**C**): distant metastasis).

When merging the results from baseline and ART simulation CTs, the AUC for predicting LR, NR, and DM occurrence was 0.773, 0.747, and 0.793 (Figure 4). In contrast, the accuracy for the three endpoints was 72.4%, 74.7%, and 75.7%, respectively.

### 3.3. Comparison with the Prediction Performance from Clinical Stage and Gross Tumor Volumes

As presented in Appendix A.2, both the GTVp (AUC = 0.617, *p* = 0.014) and clinical stage T2 versus T3–4 (AUC = 0.620, *p* = 0.011) demonstrated an association with LR. In contrast, clinical stage N0–1 versus N2–3 did not significantly correlate with the incidence of NR (AUC = 0.537, *p* = 0.49). Notably, the gross tumor volume of metastatic lymph nodes (GTVn) (AUC = 0.712, *p* = 0.001) was linked to NR. Furthermore, GTVn (AUC = 0.651, *p* = 0.021) was also associated with DM development. When patients were categorized based on the median GTVp value of 13.8 mL, those with volumes below the median exhibited a 3-year local relapse-free survival rate of 73%, compared to 60% for those with volumes above the median (*p* = 0.022). Similarly, stratifying the cohort by the median GTVn value of 6.8 mL resulted in 3-year nodal relapse-free survival rates of 88% for the group below the median and 62% for the group above (*p* = 0.001). Using the same GTVn stratification, the 3-year distant metastasis-free survival rates were 95% and 78% for the lower and higher groups, respectively (*p* = 0.021).

## 4. Discussion

ART is commonly classified into anatomy-ART and response-ART (R-ART). There is no universal agreement on the optimal methodology or definitive clinical advantages for the routine application of ART in HNSCC patients [2,5]. Recent advances in multi-modal imaging analysis have shown promising results. Yoon et al. [15] demonstrated that FDG PET radiomics features, particularly gray-level non-uniformity for zone (GLNU_GLZLM), served as an independent prognostic factor for overall survival in HNSCC patients. Similarly, Higgins et al. [16] found that pretreatment tumor SUVmean had superior prognostic value compared to SUVmax for disease-free survival. Wang et al. [17] further showed that combining PET/CT radiomics with dosiomics features and DVH parameters could achieve C-indices up to 0.873 for predicting overall survival. Nonetheless, due to the considerable variability in tumor volume and spatial changes across patients, ART may be particularly advantageous for those experiencing significant anatomical alterations and potentially improve dosimetric accuracy [5]. In contrast, R-ART can provide promise in leveraging innovative technologies to enhance precision medicine. This study uniquely demonstrated that RT-based treatment outcomes can be effectively predicted by integrating pretreatment and ART simulation CT scans, utilizing segmentation-free DL-based computer vision. Notably, the MedNeXt model employed in this study facilitated training on limited datasets and allowed for flexible adjustments in network architecture, including depth, width, and receptive field size. This adaptability enables a balanced approach between model complexity and performance, suitable for various imaging tasks.

Despite recent progress in treatment modalities, managing HNSCCs remains a complex undertaking [18]. For many patients with locally advanced oropharyngeal or hypopharyngeal cancer, organ-preserving strategies using CRT or RT are often preferred. Utilizing both baseline and ART simulation CT scans, our proposed framework showed the potential to predict the risk of LR, NR, and DM with an accuracy exceeding 70%. While promising, this level of accuracy requires further refinement for widespread clinical adoption. In DL studies focused on HNSCCs, Diamant et al. analyzed baseline CT images from the Cancer Image Archive and reported an 88% predictive accuracy for DM [11]. Similarly, Alabi et al. developed an interpretable machine learning model for OPSCC risk stratification, achieving 88.3% accuracy with voting ensemble algorithms [19]. Karadaghy et al. also demonstrated the utility of machine learning in predicting primary treatment modality for T1–2, N0-N1 OPSCC with 71% accuracy [20]. However, their model’s accuracy for locoregional failures was notably lower, at 66%. In a different context, Bonomo et al. found that radiomics features extracted from simulation CT scans of rectal cancer patients could predict favorable responses to neoadjuvant CRT, with AUC values of 0.65 in the training set and 0.63 in the validation set [21]. Future research should focus on developing optimized preprocessing techniques to enhance clinical utility across diverse endpoints. This might involve incorporating some clinical data, such as GTV, stage, or other relevant clinical parameters.

Recent advances in deep learning have shown significant promise in head and neck cancer imaging. Illimoottil and Ginat [22] comprehensively reviewed deep learning applications in MRI, CT, and PET imaging for head and neck cancers, demonstrating superior performance in tumor detection, segmentation, and outcome prediction compared to traditional methods. Advanced techniques such as convolutional autoencoders, GANs, and transformer models have further enhanced imaging capabilities, which could complement our DCL approach for improved prognostic modeling.

Although this study pioneered the DL-based prediction from both baseline and ART simulation CTs, there are two plausible reasons for the weakness of the model’s performance. First, regardless of employing the Batch Balance Wrapper framework, the impact of the imbalanced events between objects with or without the events should be further clarified. Future studies should consider maneuvering the amount of sampling data. The other reason was that the model did not include additional clinical or biological information, and the detectable DL change between the two simulation CT images might not provide sufficient biological information to foresee the events. The effective radius (ER) approach has been validated for determining optimal ART timing, as demonstrated by Albosaabar et al., who found fraction 14 optimal for small PTV volumes and fractions 7 and 21 for larger volumes [23]. To gain more information, Li et al. [24] The integration of multi-modal data has been comprehensively reviewed by Abid et al. [25], who emphasized how deep learning can combine radiomics, genomics, and clinical data to overcome the limitations of traditional diagnostic methods in capturing tumor heterogeneity. designed a longitudinal radiomic trend framework using daily CBCT scans to achieve a more robust treatment response assessment in patients with rectal cancer receiving neoadjuvant CRT. In addition, if the integration of some molecular images, such as 18F-FDG PET/CT, could be available, the overall prediction performance might be optimized further.

The importance of multi-modality fusion in improving prediction accuracy has been demonstrated in recent studies. Javanmardi et al. [26] achieved remarkable accuracy (93% for OS, 95% for DM) by combining PET-CT fusion techniques with deep learning in HNSCC patients. Their findings using fusion methods like DWT and RLPP align with our approach of integrating baseline and ART CT images, suggesting that temporal and multi-modal fusion strategies can significantly enhance prognostic accuracy.

While the results of our study are promising, it is essential to acknowledge the limitations inherent in a single-center, retrospective analysis. To ensure broader applicability and robustness, our findings require rigorous external validation using multi-center datasets and diverse imaging protocols. As highlighted in recent research on feature extraction harmonization [27], variations in image reconstruction techniques and the timing of ART simulation CTs could influence imaging data. These factors need further investigation.

Machine learning approaches have also been successfully applied to nasopharyngeal cancer prognosis. Akcay et al. [28] evaluated six different machine learning algorithms for NC survival prediction, achieving 88% accuracy with Gaussian Naive Bayes algorithm. They identified key prognostic factors including NLR, LDH, and hemoglobin levels, which supports the importance of incorporating clinical biomarkers alongside imaging features in predictive models.

Future studies could enhance predictive accuracy by incorporating a multi-institutional approach, integrating a broader range of clinical and imaging parameters, and a consistent timing of ART simulation. Additionally, the employment of image-generative artificial intelligence can produce large amount of unannotated image data, which facilitates multiple downstream DL work [29].

Despite these limitations, our study possesses several strengths. These include using consistent treatment protocols across the patient cohort and implementing novel techniques designed to minimize the risk of overfitting the model to the training data.

The findings presented have the potential to enable personalized treatment strategies for CRT or RT tailored to the specific biological characteristics of each patient’s tumor. After successful external validation, it would be prudent to consider modifying treatment approaches or exploring dose escalation for patients whose tumors exhibit these unfavorable imaging characteristics.

The feasibility of online adaptive radiotherapy has been recently demonstrated by Yoon et al. [30], who evaluated a semi-automated online ART system achieving a median planning duration of 19.6 min. While their system focused on real-time adaptation during treatment, our approach leverages the temporal changes captured between baseline and mid-treatment imaging to improve prognostic modeling.

## 5. Conclusions

When applied to patients with oropharyngeal or hypopharyngeal cancer about to undergo RT or CRT, our DL framework, leveraging both baseline and ART simulation CT scans, demonstrated promising results in predicting treatment outcomes. This was especially true for DM. Precisely, the AUC values for predicting LR, NR, and DM were 0.773, 0.747, and 0.793, respectively. The corresponding accuracies for these three endpoints were 72.4%, 74.7%, and 75.7%. While these results are encouraging, there is a clear need to enhance accuracy further by incorporating additional imaging modalities or clinical data. Furthermore, external validation using independent datasets is crucial to confirm and solidify the model’s robustness.

## Figures and Tables

**Table 1:** Patient characteristics of training cohort (*n* = 162).

Variables	*n* (%)
**Gender**	
Male	156
Female	6
**Age (year)**	median 53; range 37 to 82
**Primary tumor site**	
oropharynx	80 (49.40%)
hypopharynx	82 (50.6%)
**ECOG performance status**	
0	24 (14.8%)
1	136 (84.0%)
2	2 (1.2%)
**T classification**	
T1	11 (6.8%)
T2	53 (32.7%)
T3	42 (25.9%)
T4	56 (34.6%)
**N classification**	
N0	3 (1.9%)
N1	34 (21.0%)
N2	117 (72.2%)
N3	8 (4.9%)
**AJCC stage**	
III	23 (14.2%)
IVA	125 (77.2%)
IVB	14 (8.6%)
**Smoking**	
Smoker	138 (84.1%)
Never-smoker	24 (15.9%)
**Betel nut squid**	
Yes	85 (51.9%)
Never	77 (48.1%)
**Alcohol drinking**	
Alcoholism	74 (45.7%)
Non-alcoholism	88 (54.3%)
**Radiation dose (Gy)**	median 70.0 Gy (range, 68.4–72.0 Gy)
**Concurrent drug regimen**	
Tri-weekly cisplatin	131 (80.9%)
Cetuximab	24 (14.8%)
None	7 (4.3%)
Median follow-up durations (months)	34 (range, 6 to 158)

Abbreviation: ECOG = Eastern Cooperative Oncology Group; AJCC: American Joint Committee on Cancer.

**Table 2:** Prediction performance for local recurrence.

	TEST	AUC	Accuracy	Sensitivity	Specificity
**Baseline simulation CT**					
	K1	0.572	0.588	0.545	0.667
	K2	0.658	0.625	0.619	0.636
	K3	0.788	0.75	0.762	0.727
	K4	0.697	0.6885	0.619	0.818
	K5	0.779	0.688	0.619	0.818
	Mean	0.699	0.668	0.633	0.733
**Adaptive simulation CT**					
	K1	0.674	0.559	0.545	0.583
	K2	0.71	0.688	0.667	0.727
	K3	0.662	0.531	0.429	0.727
	K4	0.658	0.594	0.524	0.727
	K5	0.71	0.656	0.667	0.636
	Mean	0.683	0.606	0.566	0.680
**Merged ensemble model**					
	K1	0.758	0.618	0.591	0.667
	K2	0.74	0.781	0.857	0.636
	K3	0.814	0.75	0.667	0.909
	K4	0.805	0.75	0.857	0.545
	K5	0.749	0.719	0.667	0.818
	Mean	0.773	0.724	0.728	0.715

**Table 3:** Prediction performance for nodal relapse.

	TEST	AUC	Accuracy	Sensitivity	Specificity
**Baseline simulation CT**					
	K1	0.6	0.636	0.68	0.5
	K2	0.725	0.667	0.64	0.75
	K3	0.583	0.563	0.56	0.571
	K4	0.731	0.656	0.64	0.714
	K5	0.537	0.563	0.56	0.571
	Mean	0.635	0.617	0.616	0.621
**Adaptive simulation CT**					
	K1	0.745	0.667	0.68	0.625
	K2	0.61	0.515	0.48	0.625
	K3	0.686	0.656	0.64	0.714
	K4	0.8	0.656	0.6	0.857
	K5	0.606	0.719	0.8	0.429
	Mean	0.689	0.643	0.64	0.65
**Merged ensemble model**					
	K1	0.795	0.879	1	0.5
	K2	0.69	0.667	0.68	0.625
	K3	0.709	0.781	0.84	0.571
	K4	0.851	0.844	0.88	0.714
	K5	0.691	0.563	0.52	0.714
	Mean	0.747	0.747	0.784	0.625

**Table 4:** Prediction performance for distant metastasis.

	TEST	AUC	Accuracy	Sensitivity	Specificity
**Baseline simulation CT**					
	K1	0.629	0.697	0.75	0.4
	K2	0.843	0.788	0.786	0.8
	K3	0.7	0.727	0.714	0.8
	K4	0.795	0.781	0.786	0.75
	K5	0.787	0.581	0.519	1
	Mean	0.751	0.715	0.711	0.75
**Adaptive simulation CT**					
	K1	0.586	0.727	0.786	0.4
	K2	0.65	0.848	0.929	0.4
	K3	0.821	0.788	0.821	0.6
	K4	0.643	0.750	0.786	0.5
	K5	0.63	0.742	0.741	0.75
	Mean	0.666	0.771	0.812	0.53
**Merged ensemble model**					
	K1	0.814	0.848	0.893	0.6
	K2	0.857	0.848	0.857	0.8
	K3	0.757	0.697	0.679	0.8
	K4	0.75	0.750	0.750	0.75
	K5	0.787	0.645	0.630	0.75
	Mean	0.793	0.758	0.762	0.74

## Data Availability

All available data are presented in the text of the paper.

## Appendix A

### Appendix A.1. Supervised Contrastive Loss Function and Model Optimization Strategy

For model training, we employed the supervised contrastive loss function in conjunction with the Adam optimizer, initialized with a learning rate of 2 × 10^−5^. To mitigate overfitting, we implemented an early stopping protocol and utilized k-fold cross-validation (*k* = 5) for robust performance assessment. The training process was conducted with a batch size of 2, optimized for our computational resources and dataset characteristics.

The supervised contrastive loss is a loss function used for classification tasks. Its core idea is to pull samples of the same class closer together in the feature space while pushing samples of different classes further apart. To enhance the model’s performance further, this study integrated imaging features from both baseline and adaptive stages into contrastive learning by calculating the similarity between one sample and other samples within the same class and across different classes, regardless of whether the target samples came from baseline or adaptive stages. Then, by minimizing the loss function, the model could learn more comprehensive and discriminative feature representations. As a result, classification performance could be improved, especially when dealing with overlapping classes or uneven data distributions. This approach that integrates imaging features from multiple time points also enhanced the robustness of the model, making it able to handle noise and variations in the data.

## References

[1] Glide-Hurst C.K., Lee P., Yock A.D., Olsen J.R., Cao M., Siddiqui F., Parker W., Doemer A., Rong Y., Kishan A.U. (2021). Adaptive Radiation Therapy (ART) Strategies and Technical Considerations: A State of the ART Review From NRG Oncology. Int. J. Radiat. Oncol. Biol. Phys..

[2] Nuyts S., Bollen H., Eisbruch A., Strojan P., Mendenhall W.M., Ng S.P., Ferlito A. (2024). Adaptive radiotherapy for head and neck cancer: Pitfalls and possibilities from the radiation oncologist’s point of view. Cancer Med..

[3] Siegel R.L., Miller K.D., Wagle N.S., Jemal A. (2023). Cancer statistics, 2023. CA Cancer J. Clin..

[4] Atun R., Jaffray D.A., Barton M.B., Bray F., Baumann M., Vikram B., Hanna T.P., Knaul F.M., Lievens Y., Lui T.Y. (2015). Expanding global access to radiotherapy. Lancet Oncol..

[5] Morgan H.E., Sher D.J. (2020). Adaptive radiotherapy for head and neck cancer. BMC Cancer.

[6] Gan Y., Langendijk J.A., Oldehinkel E., Lin Z., Both S., Brouwer C.L. (2024). Optimal timing of re-planning for head and neck adaptive radiotherapy. Radiother. Oncol..

[7] Kumar B., Cordell K.G., Lee J.S., Worden F.P., Prince M.E., Tran H.H., Wolf G.T., Urba S.G., Chepeha D.B., Teknos T.N. (2008). EGFR, p16, HPV titer, Bcl-xL and p53, sex, and smoking as indicators of response to therapy and survival in oropharyngeal cancer. J. Clin. Oncol..

[8] Zhang J., Lam S.K., Teng X., Ma Z., Han X., Zhang Y., Cheung A.L., Chau T.C., Ng S.C., Lee F.K. (2023). Radiomic feature repeatability and its impact on prognostic model generalizability: A multi-institutional study on nasopharyngeal carcinoma patients. Radiother. Oncol..

[9] Lou B., Doken S., Zhuang T., Wingerter D., Gidwani M., Mistry N., Ladic L., Kamen A., Abazeed M.E. (2019). An image-based deep learning framework for individualizing radiotherapy dose. Lancet Digit. Health.

[10] Fujima N., Andreu-Arasa V.C., Meibom S.K., Mercier G.A., Truong M.T., Hirata K., Yasuda K., Kano S., Homma A., Kudo K. (2021). Prediction of the local treatment outcome in patients with oropharyngeal squamous cell carcinoma using deep learning analysis of pretreatment FDG-PET images. BMC Cancer.

[11] Diamant A., Chatterjee A., Vallières M., Shenouda G., Seuntjens J. (2019). Deep learning in head & neck cancer outcome prediction. Sci. Rep..

[12] Huynh B.N., Groendahl A.R., Tomic O., Liland K.H., Knudtsen I.S., Hoebers F., van Elmpt W., Malinen E., Dale E., Futsaether C.M. (2023). Head and neck cancer treatment outcome prediction: A comparison between machine learning with conventional radiomics features and deep learning radiomics. Front. Med..

[13] Yang S.N., Liao C.Y., Chen S.W., Liang J.A., Tsai M.H., Hua C.H., Lin F.J. (2011). Clinical implications of the tumor volume reduction rate in head-and-neck cancer during definitive intensity-modulated radiotherapy for organ preservation. Int. J. Radiat. Oncol. Biol. Phys..

[14] Giorgi J., Nitski O., Wang B., Bader G. (2006). Deep contrastive learning for unsupervised textual representations. arXiv.

[15] Yoon H., Ha S., Kwon S.J., Park S.Y., Kim J., O J.H., Yoo I.R. (2021). Prognostic value of tumor metabolic imaging phenotype by FDG PET radiomics in HNSCC. Ann. Nucl. Med..

[16] Higgins K.A., Hoang J.K., Roach M.C., Chino J., Yoo D.S., Turkington T.G., Brizel D.M. (2012). Analysis of pretreatment FDG-PET SUV parameters in head-and-neck cancer: Tumor SUVmean has superior prognostic value. Int. J. Radiat. Oncol. Biol. Phys..

[17] Wang B., Liu J., Zhang X., Wang Z., Cao Z., Lu L., Lv W., Wang A., Li S., Wu X. (2023). Prognostic value of 18F-FDG PET/CT-based radiomics combining dosiomics and dose volume histogram for head and neck cancer. EJNMMI Res..

[18] Argiris A., Karamouzis M.V., Raben D., Ferris R.L. (2008). Head and neck cancer. Lacent.

[19] Alabi R.O., Almangush A., Elmusrati M., Leivo I., Mäkitie A.A. (2022). An interpretable machine learning prognostic system for risk stratification in oropharyngeal cancer. Int. J. Med. Inform..

[20] Karadaghy O.A., Shew M., New J., Bur A.M. (2021). Machine Learning to Predict Treatment in Oropharyngeal Squamous Cell Carcinoma. ORL.

[21] Bonomo P., Socarras Fernandez J., Thorwarth D., Casati M., Livi L., Zips D., Gani C. (2022). Simulation CT-based radiomics for prediction of response after neoadjuvant chemo-radiotherapy in patients with locally advanced rectal cancer. Radiat. Oncol..

[22] Illimoottil M., Ginat D. (2023). Recent Advances in Deep Learning and Medical Imaging for Head and Neck Cancer Treatment: MRI, CT, and PET Scans. Cancers.

[23] Albosaabar M.H., Ahmad R.B., Abouelenein H., Mohamed F., Yahya N., Mohamed D.O., Sayed M.M. (2024). Determining The Optimal Time To Apply Adaptive Radiotherapy Plan For Head And Neck Cancer Patients. J. Pioneer. Med. Sci..

[24] Li Z., Raldow A.C., Weidhaas J.B., Zhou Q., Qi X.S. (2023). Prediction of radiation treatment response for locally advanced rectal cancer via a longitudinal trend analysis framework on cone-beam CT. Cancer.

[25] Abid N., Neoaz N., Amin M.H. (2024). Deep learning for multi-modal cancer imaging: Integrating radiomics, genomics, and clinical data for comprehensive diagnosis and prognosis. Glob. J. Univers. Stud..

[26] Javanmardi A., Hosseinzadeh M., Hajianfar G., Nabizadeh A.H., Rezaeijo S.M., Rahmim A., Salmanpour M. (2022). Multi-modality fusion coupled with deep learning for improved outcome prediction in head and neck cancer. Proc. SPIE.

[27] Toosi A., Shiri I., Zaidi H., Rahmim A. (2024). Segmentation-free outcome prediction from head and neck cancer PET/CT images: Deep learning-based feature extraction from Multi-Angle Maximum Intensity Projections (MA-MIPs). Cancer.

[28] Akcay M., Etiz D., Celik O., Ozen A. (2020). Evaluation of Prognosis in Nasopharyngeal Cancer Using Machine Learning. Technol. Cancer Res. Treat..

[29] Jung H.K., Kim K., Park J.E., Kim N. (2024). Image-based generative artificial intelligence in radiology: Comprehensive updates. Korean J. Radiol..

[30] Yoon S.W., Lin H., Alonso-Basanta M., Anderson N., Apinorasethkul O., Cooper K., Dong L., Kempsey B., Marcel J., Metz J. (2020). Initial Evaluation of a Novel Cone-Beam CT-Based Semi-Automated Online Adaptive Radiotherapy System for Head and Neck Cancer Treatment—A Timing and Automation Quality Study. Cureus.

